# Treatment of Hyperammonemia Syndrome in Lung Transplant Recipients

**DOI:** 10.3390/jcm12226975

**Published:** 2023-11-08

**Authors:** Sarah Yun, Ciana Scalia, Sara Farghaly

**Affiliations:** 1 The Mount Sinai Hospital, New York, NY 10029, USA; ciana.scalia@mountsinai.org; 2 Montefiore Medical Center, Bronx, NY 10467, USA; sfarghal@montefiore.org

**Keywords:** hyperammonemia, lung transplant, Ureaplasma, Mycoplasma, infection, protein, amino acids, parenteral nutrition

## Abstract

Hyperammonemia syndrome is a complication that has been reported to occur in 1–4% of lung transplant patients with mortality rates as high as 60–80%, making detection and management crucial components of post-transplant care. Patients are treated with a multimodal strategy that may include renal replacement therapy, bowel decontamination, supplementation of urea cycle intermediates, nitrogen scavengers, antibiotics against *Mollicutes*, protein restriction, and restriction of parenteral nutrition. In this review we provide a framework of pharmacologic mechanisms, medication doses, adverse effects, and available evidence for commonly used treatments to consider when initiating therapy. In the absence of evidence for individual strategies and conclusive knowledge of the causes of hyperammonemia syndrome, clinicians should continue to design multimodal regimens based on suspected etiologies, institutional drug availability, patient ability to tolerate enteral medications and nutrition, and availability of intravenous access.

## 1. Introduction

Hyperammonemia syndrome is a complication that has been reported to occur in 1–4% of lung transplant patients with mortality rates as high as 60–80%, making detection and management crucial components of post-transplant care [1,2,3]. There is no standardized treatment algorithm, nor is there any research comparing the various options for this indication. Most simultaneously employ a variety of therapies in hopes of controlling this potentially fatal condition, and though there are common themes throughout published cases, comprehensive details for each therapy are often not provided. In this review we aim to describe treatment strategies for hyperammonemia syndrome after lung transplant in more detail and provide a framework for clinicians initiating therapy.

## 2. Pathophysiology

The majority of natural ammonia production in the human body occurs in the gut after protein metabolism. These nitrogenous waste products are eliminated by conversion to urea through the urea cycle in the liver and subsequently excreted by the kidneys, or by conversion to glutamine in the liver, skeletal muscle, and central nervous system [2,4,5]. Acute hyperammonemia, most commonly presenting as encephalopathy, can result when mechanisms of elimination are disrupted or when there is excessive production. Seizures in the setting of encephalopathy may occur when excess glutamine causes astrocyte swelling and excessive glutamatergic activity due to glutamate receptor downregulation [6]. The exact cause of hyperammonemia syndrome in the lung transplant population is unknown but may involve multiple processes.

It has been proposed that the physiological stress of lung transplant may unmask previously undiagnosed urea cycle disorders (UCD). Kamel et al. were unable to find cases of lung transplant patients with UCDs in their systematic review, but they did find evidence to support downregulation of hepatic glutamine synthetase in lung transplant patients. This may result in decreased ammonia elimination [5]. Malnutrition is rarely investigated in the context of hyperammonemia after lung transplant but has been linked with higher prevalence of non-hepatic hyperammonemia in other populations [7,8,9,10]. Poor nutrition status can lead to functional deficiencies of substances necessary for ammonia detoxification. A prospective study of non-hepatic hyperammonemia in critically ill patients by Prado et al. found those who developed hyperammonemia had received less calories per day (as a total of enteral nutrition or parenteral nutrition (PN)) and that hyperammonemia was statistically associated with prolonged fasting for 24 h or more (*p* < 0.05) [8]. Other possible mechanisms of decreased ammonia clearance post-transplant include immunosuppression and stress-induced loss of muscle mass as well as acute kidney injury [2,6,11].

In addition to immediate post-transplant complications that can disrupt ammonia metabolism, bacteria from the class *Mollicutes* have been a significant contributor to post-lung transplant hyperammonemia. Buzo et al. encountered 7 cases of hyperammonemia syndrome in their lung transplant recipients between 2012 and 2019, and all tested positive for *Mollicutes*. Also, of the 49 cases of hyperammonemia identified in their literature review 18 patients had specimens sent for testing and 16 were positive for *Mollicutes* [1]. *Ureaplasma urealyticum*, *Mycoplasma hominis*, *and Ureaplasma parvum* are *Mollicutes* commonly found in the urogenital and sometimes the upper respiratory tract but may be present in other areas of the body in the setting of immunosuppression and broad-spectrum antimicrobials [1,2,3,4,12]. Through different mechanisms, these species are able to generate ammonia. *Ureaplasma parvum* and *urealyticum* hydrolyze urea into ammonia and carbon dioxide, and their presence in both donors and recipients have been associated with higher ammonia levels in lung transplant recipients [3,4,6,13]. *Mycoplasma hominis* depletes arginine to generate energy, which decreases the arginine available to clear ammonia through the urea cycle. Multiple cases have been reported of patients infected with *Mycoplasma hominis*, but patients are often co-infected with *Ureaplasma* species so *Mycoplasma’s* independent role in the clinical development of hyperammonemia syndrome is less clear [3,6,14].

## 3. Detection and Diagnosis

Diagnosis of hyperammonemia syndrome usually occurs within the first 2 weeks after transplant, though the criteria for diagnosis is not standardized [1]. It is possible for the diagnosis to be delayed after a lung transplant due to its overlapping signs and symptoms with other conditions, such as intensive care unit delirium, concomitant infections, use of sedation, and side effects of immunosuppression. Blood ammonia levels, liver function tests, tests for urea cycle disorders, imaging, and antimicrobial cultures and molecular testing can help identify hyperammonemia syndrome and guide treatment. Kamel et al. routinely check daily ammonia levels for the first week after lung transplant as well as after any change in mental status, and they found that their strategies allowed for earlier detection of hyperammonemia and potentially lowered mortality [5]. There is no universally accepted ammonia level threshold for the diagnosis of hyperammonemia syndrome, but some examples in the literature include ammonia greater than 50, 60, 90, or 200 µmoL/L [1,2,4,5,6,15]. Reported peak levels also vary widely ranging from 143 to 5000 µmol/L [1,16]. In addition to ammonia levels, published reports have included various components in their diagnostic criteria to distinguish clinically significant hyperammonemia such as absence of hepatic abnormalities, encephalopathy, administration of specific treatment for hyperammonemia, and time from transplant (e.g., within 30 or 45 days of transplant) [1,5,6]. Imaging studies may be unremarkable or reveal a spectrum of findings including cerebral edema [1]. The detection of *Mollicutes* is more challenging. The *Ureaplasma* species require a special medium for growth and can take up to 5 days to produce results, and *Mycoplasma hominis* grows in pinpoint colonies that are easy to miss. Therefore, polymerase chain reaction (PCR) testing, most commonly done using bronchoalveolar lavage samples, can be done in conjunction [12,13,17]. As these infections can be donor-derived, testing of donors may also be considered [3].

## 4. Management

Data for the management of hyperammonemia syndrome in this population is limited to case reports and case series and there are no comparative studies for the various treatment strategies. A multimodal strategy involving direct removal of ammonia, increasing clearance, and decreasing production is usually used [1,6].

### 4.1. Renal Replacement Therapy

Renal replacement therapy is used to rapidly remove ammonia from the circulation, and intermittent hemodialysis is the most efficient modality though there can be a rebound in ammonia levels afterwards [18]. Continuous renal replacement therapy removes ammonia more slowly but causes less intradialytic hypotension and may be preferred in patients with hemodynamic instability. It can also be used after sessions of intermittent hemodialysis to prevent rebound, since it allows for continuous ammonia removal [11]. Peritoneal dialysis is not effective and not recommended [19,20]. Lastly, charcoal column hemoperfusion does not appear to be helpful [18,19,20].

### 4.2. Bowel Decontamination

Bowel decontamination has been widely used to increase ammonia elimination through the colon or decrease urea-producing enteric bacteria, though evidence for its benefit in this setting is lacking. Lactulose, rifaximin, and metronidazole were primarily studied in chronic hepatic encephalopathy, although lactulose specifically has been shown to have no effect on mortality in acute hyperammonemia [2,11,21,22]. Lactulose is a nonabsorbable disaccharide broken down by bacteria in the colon into lactic, formic, and acetic acids, resulting in an acidic pH and conversion of ammonia to ammonium, which cannot diffuse into the bloodstream. The acidic pH may also be less hospitable to ammonia-producing bacteria [11]. It acts as a laxative by increasing osmotic pressure in the intestine which results in excess water being pulled in and fecal excretion of ammonia [23]. It is supplied as 10 and 20 g packets to be dissolved in water as well as a 10 g per 15 mL oral solution. Doses range from 10–30 g orally (PO) 2–4 times daily and can be titrated to obtain adequate bowel movements [4,15]. Use may be limited by gastrointestinal symptoms such as diarrhea, abdominal pain, nausea, and flatulence [23].

Antibiotics have also been used to decrease ammonia-forming bacteria in the gut. Metronidazole is a nitroimidazole antibiotic with activity against ammonia producing Gram-negative anaerobes. It interferes with DNA synthesis and degradation, leading to bacterial cell death [24]. It is available as tablets and an intravenous solution. Dosing is 500 mg PO or IV every 8 h for this indication, but adjustment to every 12 h dosing may be needed for severe hepatic impairment [5,15,24]. Adverse effects include gastrointestinal effects and neurotoxicity [24]. Rifaximin is a rifamycin antibiotic that binds to bacterial DNA-dependent RNA polymerase, inhibiting bacterial protein synthesis and growth. It is available in tablet form and dosed at 550 mg PO every 12 h [4,5,15,25]. Adverse effects may include nausea, flatulence, diarrhea, peripheral edema, and dizziness [11,25]. Generic formulations are not available, and therefore access may be a barrier. Neomycin has been used in the past but can result in ototoxicity and renal failure [15].

### 4.3. Supplementation of Urea Cycle Intermediates

Levocarnitine supplementation has been used to decrease ammonia levels and improve mental function. Carnitine is involved in uptake of fatty acids by mitochondria and the production of coenzyme A and is an intermediate of the urea cycle. It has been proposed to prevent protein catabolism and stimulate the urea cycle to increase clearance of ammonia via ureagenesis [2,26]. In studies of mice injected with ammonium acetate and levocarnitine, levocarnitine administration decreased brain and blood ammonia levels in addition to preventing symptoms such as drowsiness and seizures [27]. Administration details in human lung transplant recipients such as route, dose, and frequency vary between publications. Treatment protocols from Chen et al. and Kamel et al. both include levocarnitine 100 mg/kg per 24 h intravenously (IV) either divided every 4 h or as a continuous infusion [5,15]. Moffatt-Bruce et al. also administered 100 mg/kg intravenously but did not specify if repeat doses were given [28]. Bharat et al. published a protocol with levocarnitine 100 mg/kg divided into 6 doses with a maximum of 300 mg/kg over 24 h. However, it was unclear if repeat doses were given and if the dosing referred to oral or intravenous formulations [4]. Another strategy seen was 100 mg/kg IV as a loading dose followed by 50 mg/kg IV every 8 h and 9800 mg IV daily (patient weight not reported) [16,18].

Levocarnitine is available as a 200 mg/mL intravenous solution, a 1 g/10 mL oral solution, and 330 mg tablets. Though most used intravenous levocarnitine in these publications with lung transplant patients and the oral bioavailability is low at around 15%, there is literature to support oral levocarnitine’s ability to lower ammonia levels in patients with hepatic encephalopathy and valproic acid-induced hyperammonemia. Doses used were 2 g PO twice daily for hepatic encephalopathy and a median of 990 mg per day PO for valproic acid-induced hyperammonemia [26,29,30]. Reports of adverse effects due to levocarnitine were not common, but some gastrointestinal effects and headache were noted in the hepatic encephalopathy studies. Slowing consumption (of oral solution), dividing doses throughout the day, and decreasing doses may improve gastrointestinal side effects. More vigilant monitoring for adverse effects may also be necessary in patients with impaired renal function as levocarnitine’s metabolites are renally eliminated [31].

Arginine, another urea cycle intermediate, has also been used to prevent protein catabolism and facilitate the urea cycle [21]. Arginine supplementation is available as an arginine hydrochloride 10% intravenous solution under the brand name R-Gene 10 and can be combined with an infusion of sodium phenylacetate and sodium benzoate (discussed below) [32,33]. Reported dosing of arginine also varies. Chen et al. administered 200 mg/kg IV over 90 min, followed by 200 mg/kg IV daily over 24 h continuously as part of their protocol for treatment of hyperammonemia [15]. The protocol from Kamel et al. uses an initial dose of 200 mg/kg intravenously daily as a continuous infusion with the option to decrease to a maintenance dose of 4 g/m^2^ after 48 h [5]. Other published doses and frequencies have also included 21 g IV daily (patient weight not reported) and 200–600 mg/kg IV over 90 min followed by 200–600 mg/kg/day IV continuously [16,18,34]. Moffatt-Bruce et al. administered 210 mg/kg IV but did not specify if repeat doses were given [28]. Central line placement is favored for the administration of arginine due to the risk of extravasation [35]. Potential adverse effects include abdominal pain, diarrhea, and hyperchloremic metabolic acidosis that is secondary to chloride content present in R-Gene 10^®^. The excess chloride may result in a shift of bicarbonate from the extracellular space to the intracellular space in an effort to preserve equilibrium, thereby diminishing the bicarbonate available to maintain an optimal pH [36]. Additionally, in patients with renal dysfunction, the clearance of nitrogenous waste resulting from arginine breakdown may be delayed.

### 4.4. Nitrogen Scavengers

Nitrogen scavengers benzoate, phenylacetate, and phenylbutyrate are commonly added to treatment regimens to help decrease ammonia levels. Benzoate conjugates with L-glycine and phenylacetate conjugates with glutamine. Both processes form nitrogen-containing products that are then eliminated and have also been shown to decrease blood ammonia levels [37]. Phenylbutyrate is a prodrug that is oxidized to phenylacetate in the liver, and glycerol phenylbutyrate is a prodrug formulation that requires pancreatic enzymes to release phenylbutyrate [37,38].

Sodium phenylacetate and sodium benzoate are available as a combined intravenous product. It facilitates faster nitrogen removal than sodium benzoate alone through the use of two conjugation pathways [39]. It is available as a brand name product, Ammonul^®^, and as a generic. Both are 10–10% solutions containing 100 mg of sodium acetate and 100 mg of sodium benzoate per milliliter. A central line must be used for administration to avoid burning, and the product must be diluted with at least 25 mL/kg of 10% dextrose [33]. Dosing regimens seen in the literature include a loading dose of 250 mg/kg IV followed by 250 mg/kg IV daily or 5.5 g/m^2^ IV over 90 min followed by 5.5 g/m^2^/day IV continuously [16,34]. Oral sodium benzoate had similar efficacy when a dose of 5 g twice daily was compared to lactulose for acute portal-systemic encephalopathy, but it is only available alone as a powder that must be compounded into formulations that are able to be administered. Kamel et al. added 5.5 g/m^2^ PO daily divided every 6 h if ammonia was not controlled after 72 h of sodium phenylbutyrate for more aggressive enteral clearance as a less costly alternative to intravenous sodium phenylacetate and sodium benzoate [5]. Side effects due to the combination of sodium benzoate and sodium phenylacetate were not commonly found in lung transplant reports, but possibilities include hypokalemia, fluid overload, neurotoxicity, and metabolic acidosis [33].

Glycerol phenylbutyrate and the oral formulations of sodium phenylbutyrate are not indicated for the treatment of acute hyperammonemia and carry warnings from manufacturers to utilize more rapidly acting interventions in these cases [37,38]. However, they are included in the multimodal approaches published by Chen et al., Bharat et al., and Kamel et al. [4,5,15]. Sodium phenylbutyrate is available in a variety of enteral dosage forms including packet for suspension (Olpruva^®^), pellets (Pheburane^®^), powder (Buphenyl^®^, generic), and tablets (Buphenyl^®^, generic). Dosing is 9.9–20 g/m^2^/day, administered in equally divided doses 3–6 times per day [5,15,40,41,42]. Glycerol phenylbutyrate is supplied as a 1.1 g/mL oral liquid and is given in 3 divided doses per day at doses ranging from 5–12.4 mL/m^2^/day [4,38]. Sodium phenylbutyrate may cause decreased appetite, taste disturbances, body odor, and neurotoxicity [11]. Glycerol phenylbutyrate has better tolerability compared to sodium phenylbutyrate with improved taste and avoidance of the high sodium content of sodium phenylbutyrate [37]. However, it may not be the optimal choice for all patients as it is a weak CYP3A4 inducer and requires adequate pancreatic enzymes for activation [38].

### 4.5. Antibiotics

Systemic antibiotics may be required to treat those who have hyperammonemia syndrome due to infections with *Mollicutes* to target the source of excess ammonia production, as routine perioperative antibiotics do not cover these organisms. Macrolides are not active against *Mycoplasma hominis* but can be used for *Ureaplasma.* Clindamycin is not active against *Ureaplasma* but is against *Mycoplasma hominis.* Tetracyclines and fluoroquinolones are active against both species. Concerns for resistance are present for all active agents and therefore empiric combination therapy can be considered while performing sensitivity testing or identifying resistance-determining gene mutations [4,6,12]. Examples of reported antibiotic regimens include azithromycin 500 mg PO/IV daily, levofloxacin 500 or 750 mg PO/IV daily, which should be renally adjusted, doxycycline 100 mg PO/IV every 12 h, and moxifloxacin 400 mg PO/IV daily [5,16]. These agents may cause gastrointestinal adverse effects, the QT interval should be monitored with both azithromycin and fluoroquinolones especially in combination with post-transplant medication such as calcineurin inhibitors and azole antifungals, and the risk of *Clostridioides difficile* infection is increased with clindamycin and fluoroquinolones. Duration of antibiotic treatment varies but ranges from two to four weeks [3,12]. There have been multiple strategies for identifying candidates for antibiotic treatment in the literature given the difficulty and time required to diagnose these infections, but there is no consensus on the optimal method. 

Roberts et al. studied multiple strategies such as testing all candidates for *Ureaplasma* species in the urine and saliva and treating positive participants for 14 days prior to transplant, empiric treatment of all donors prior to procurement, treating recipients who received an organ from a *Ureaplasma* positive donor, and treating all recipients from postoperative day 0 until donor testing returned negative. This study did not report any antimicrobial-related adverse effects, but concluded there is little role for treating colonized transplant candidates prior to transplant. Instead, screening donors for *Ureaplasma* and treating recipients early can reduce the risk of hyperammonemia syndrome. Their results included only a small pool of positive donors and should be confirmed with additional studies to determine whether there is an impact on outcomes such as mortality [3]. Alternatively, some initiate antibiotic treatment empirically in acutely hyperammonemic patients while waiting for *Mollicute* test results given the difficulty of identifying these organisms and the prevalence of infection in this population [1,6].

### 4.6. Protein Restriction

Protein restriction has historically been employed in hyperammonemia of various different etiologies as a means to reduce available substrate for ammoniagenesis and is also commonly found in reports of hyperammonemia syndrome after lung transplant. This started with hepatic encephalopathy as early as the 1950′s [43]. However, more recent research has found restricting protein intake actually increases protein breakdown while high calorie/protein intakes improve outcomes, including reduced ammonia levels [44,45]. There are no existing trials to date directly investigating the relationship between protein intake and ammonia levels in lung transplant. Current data on protein restriction in hyperammonemia after lung transplant is limited to case reports/series where specific details on protein and/or nutrition are scarce and outcomes are confounded by the muliti-modal approach uniformly employed [1,5,6,15,46]. Based on the hypothesis of an underlying UCD driving the hyperammonemia post-transplant, many have adopted strategies from UCD treatment protocols to eliminate protein for 24–48 h while infusing IV lipid and dextrose solutions to achieve 110% estimated energy requirement with a goal of preventing further amino acid catabolism, stimulating ureagenesis, and promoting anabolism [5,15,28,46,47,48]. However, UCDs are exceedingly rare, and testing for such frequently shows negative in both lung transplant and critically ill (non-liver disease) populations [1,5,7,9,49]. In a single case report of hyperammonemia after lung transplant by Moffatt-Bruce, non-protein-based nutrition was implemented and the patient was metabolically maintained on 25% dextrose and 20% lipids for a total of 1200 kcal/day. However, hyperammonemia persisted necessitating further treatment intervention with eventual recovery. No details were provided regarding protein re-introduction amount or timing [28]. A more recent study by Kamel et al. reported 3 cases of complete protein elimination for 24–48 h with IV lipid and dextrose used as calorie sources in lung transplant patients with hyperammonemia syndrome and normal systemic amino acid profiles. Similarly, ammonia continued to fluctuate despite protein removal. Protein was eventually re-introduced after 48 h at 0.25 g/kg and gradually advanced with eventual normalization in ammonia levels and survival in all 3 cases [5]. Persistent fluctuations in serum ammonia despite complete protein elimination would suggest that exogenous amino acid provision is not a primary contributor to ammoniagenesis in this setting.

Moreover, protein provides essential precursors to a variety of vital physiologic substances, for which demand is known to markedly increase following an insult such as transplant surgery. Insufficient protein intake relative to physiologic demand further exacerbates catabolism. Thus, restricting protein may result in further elevation of ammonia levels secondary to accelerated degradation of muscle and/or impaired ability to clear excess ammonia due to reduced muscle mass. Inadequate protein intake also increases risk of malnutrition which is known to be associated with worse outcomes including higher complication rates and mortality. So, while it may be tempting to limit protein intake, this does not come without significant risks and may confer net harm.

### 4.7. Parenteral Nutrition

Earlier research has linked PN, particularly the amino acid component, to hyperammonemia, thus prompting a recommendation to restrict or avoid PN. Much of the literature guiding this recommendation originates from older case reports of infants or inborn UCDs which are rare in this population [1,5,49,50,51,52,53,54]. Furthermore, many of these cases were reported during a time when common practice was to provide much higher amounts of amino acids than what is recommended today, possibly indicating that the hyperammonemia observed was simply a result of overfeeding amino acids in excess of physiologic demand. In the largest and most detailed study on the topic to date, Lichtenstein et al. described 6 cases of hyperammonemia following lung transplant while on total parenteral nutrition (TPN). There were wide variations regarding the timing of TPN initiation (post-transplant day 2–26) and amount of protein provided (0.8–2.0 g/kg/day). All cases had a major medical complication followed by elevation in ammonia after which TPN was promptly held. However, ammonia levels continued to fluctuate despite the discontinuation of TPN with eventual death in all but one. Discontinuation of TPN did not have a clear benefit in this cohort. Additionally, patients also received various combinations of ammonia-lowering therapies concurrently, confounding the ability to discern individual impact of the TPN specifically [34].

Furthermore, use of PN has traditionally been reserved for those with gastrointestinal complications rendering them unable to tolerate enteral nutrition and/or extremely poor nutrition status, thus limiting data regarding its use to an overall sicker population. Therefore, the connection between PN and hyperammonemia may merely be reflective of a highly catabolic and undernourished population rather than harm induced by the PN itself.

## 5. Conclusions and Future Directions

Hyperammonemia syndrome is a potentially fatal complication after lung transplant that lacks standardized treatment strategies. Published treatment protocols stratify options based on the severity of hyperammonemia but differ in the cutoffs and methods used. The three protocols examined during this review all use a multimodal approach, consider renal replacement therapy and protein restriction, include bowel decontamination agents as first-line pharmacologic treatment, and reserve intravenous sodium phenylacetate and sodium benzoate for patients with higher ammonia levels. In the absence of evidence for individual strategies and conclusive knowledge of the causes of hyperammonemia syndrome, clinicians should continue to design multimodal regimens based on severity, suspected etiologies, institutional drug availability, patient ability to tolerate enteral medications and nutrition, and availability of intravenous access. In this review we have provided a framework of pharmacologic mechanisms, medication doses, adverse effects, and available evidence for commonly used treatments to consider when initiating therapy. More research is needed to answer questions about the mechanisms of hyperammonemia, how to efficiently test for it, and the effectiveness of individual treatment modalities in the context of the various proposed causes.

## Data Availability

Not applicable.
